# Supplementary figures and images for: Limited variation in microbial communities across populations of Macrosteles leafhoppers (Hemiptera: Cicadellidae)
Source: Environ Microbiol Rep. 2024 Jun 10;16(3):e13279. doi: 10.1111/1758-2229.13279 (PMC11163331; doi:10.1111/1758-2229.13279)

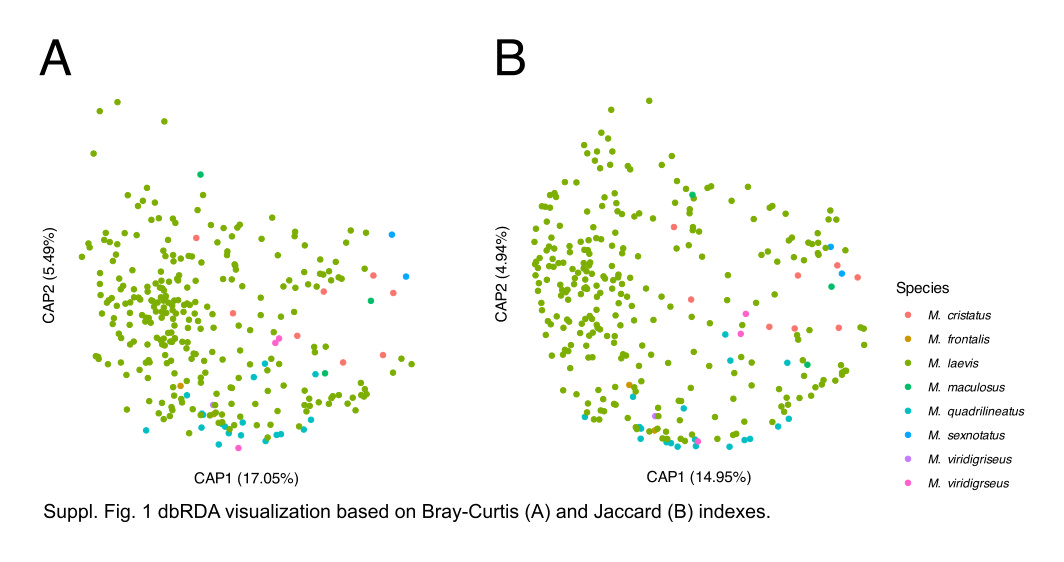

Supplement: Supplementary file 1 — Figure S1. Supplementary figure. [file EMI4-16-e13279-s002.tif]
